# Adding dandelion to rabbit diets: enhancing the growth performance and meat quality by altering immune competence and gut microbiota

**DOI:** 10.1128/spectrum.00646-25

**Published:** 2025-06-12

**Authors:** Xiaohua Yi, Xiaoqin Tang, Yuan Sui, Zhanjun Ren, Xiuzhu Sun, Shuhui Wang

**Affiliations:** 1 College of Animal Science and Technology, Northwest A&F University, Yangling, China; 2 College of Grassland Agriculture, Northwest A&F University, Yangling, China; Chengdu University, Chengdu, Sichuan, China

**Keywords:** Rabbit, Dandelion, Growth performance, Antioxidant capacity, Immune capacity

## Abstract

Healthy meat quality is an important guarantee for food safety. This study evaluated the addition of dandelion meal to the diet to investigate its effects on growth performance, carcass traits, antioxidant capacity, immune performance, and intestinal flora of Hycole meat rabbits. In the experiment, we assessed changes in meat quality, immune markers, and gut flora in rabbits by ELISA, serological assays, and 16S rRNA sequencing. The results showed that the addition of 1.5% and 2% dandelion to the diet significantly increased the daily weight gain of meat rabbits and reduced the feed-to-weight ratio (*P* < 0.05); the drip loss of the 1.5% dandelion group was significantly lower than that of the control group (*P* < 0.05); the addition of dandelion to the diet significantly reduced the content of MDA (*P* < 0.05), and had no significant effect on the blood indexes (*P* > 0.05), and the addition of 2% dandelion significantly reduced the content of gut flora (*P* > 0.05). The addition of 2% dandelion significantly decreased serum SOD content (*P* < 0.05); IgA, IgG, and IgM in the serum of meat rabbits were significantly increased by the addition of dandelion to the diet (*P* < 0.05); moreover, the analysis of the intestinal flora showed that the bacterial abundance of the group with the addition of 0.5% dandelion was the highest and significantly higher than that of the other groups (*P* < 0.05). This study provides a theoretical reference for exploring the application of Chinese herbal medicine in rabbit breeding. It provides significant value for improving the quality of rabbit meat, reducing the use of antibiotics, and providing healthy meat products for humans.

**IMPORTANCE:**

This study provides a theoretical reference for exploring the application of Chinese herbal medicine in rabbit breeding. It provides significant value for improving the quality of rabbit meat, reducing the use of antibiotics, and providing healthy meat products for humans.

## INTRODUCTION

Since the growth-promoting effect of antibiotics was discovered in 1950, antibiotics have gradually had a profound impact on global animal production. For example, they can improve feed conversion efficiency, promote animal growth, and enhance meat quality. Therefore, antibiotics have been widely used in the production of various types of animals (1). However, while antibiotics have solved the problem of disease control in factory farming with high-density stocking and have effectively improved animal production performance, they have also put great pressure on the environment (2). Antibiotics in the environment have gradually increased the drug resistance of pathogens, posing a threat to human health and safety (3). Therefore, finding alternatives to antibiotics is an important guarantee for the healthy development of animal husbandry.

Dandelion (*Taraxacum genus*) is a plant of the Asteraceae family, which is a perennial herb rich in sugar and protein with significant medicinal value. It has a complex classification, comprising over 300 species, mainly containing olefins, alkanes, alcohols, ketones, and other substances (4, 5). Taraxacum can be used as diuretics, antioxidants, bile agents, anti-inflammatory, analgesic, and anti-cancer agents (6, 7). Previous studies have found that the dandelion and its components have significant inhibitory effects against various tumor cells (8). Studies have shown that dandelion may have potential hypolipidemic and antioxidant effects based on the fact that the addition of 1% dandelion root and leaves increased plasma antioxidant enzyme activity and reduced blood lipids in cholesterol-fed rabbits (9). Past experimental studies have shown that treatments with various dandelion extracts reduce adipogenesis and lipid accumulation and decrease the severity of atherosclerosis (10). A recent study has shown that ganoderic acid A, a triterpenoid compound derived from dandelion, can reduce the accumulation of senescent cells and the physiological decline in multiple organs in prematurely aged mice induced by irradiation, naturally aged mice, and diet-induced obese mice (11).

It has been shown that the addition of dandelion to various animal feeds has an effect on their growth performance and other aspects. Dietary supplementation with dandelion improved the growth performance, serum biochemical indexes, antioxidant function, and intestinal morphology and modulated the cecal microbiota composition of Wenchang chickens (12). In a related feeding trial with pigs, the addition of dandelion powder to the feed gave similar results (13). In a comparative study on the effect of dietary mixture of herbs and dandelion as a source of probiotics on the performance of broiler chicks, it was found that the addition of a mixture of herbs or dandelion to the diet did not significantly affect the blood traits of broiler chicks and did not improve their stress coefficients, but chicks fed dandelion showed a significant increase in weight gain, feed conversion ratio, live weight, and reduced mortality (14). In the study of the effect of different levels of dandelion powder in diets on the performance and physiological performance of Ross-308 broiler chickens, it was found that the addition of 1‰ or 2‰ dandelion powder in broiler diets increased the average live weight and total body weight gain of the broilers, reduced the rate of feed consumption, increased the rate of feed conversion, lowered cholesterol, and lowered the levels of blood glucose, triglycerides, etc. and also lowered the concentration of malondialdehyde (15).

The microbial communities inhabiting the internal and external surfaces of humans and animals constitute complex ecosystems, with a total biomass far exceeding the number of host cells (16). This symbiotic system has evolved through co-evolution into a multi-dimensional interaction network, playing a central role in host metabolism, immunity, and neural regulation. It is vital to both humans and animals (17–20). In the field of animal health, rumen microbiota in ruminants synergistically secrete cellulases to convert plant fibers into volatile fatty acids, sustaining herbivorous nutritional strategies (21). A recent study has shown that *Lactobacillus salivarius* derived from chicken manure has anti-colitis effects and immunomodulatory functions (22).

At present, research on dandelion as a traditional Chinese herbal medicine additive for rabbits is still lacking. The effect of adding dandelion to the feed ration on meat rabbits is not clear. Therefore, this study aims to analyze and investigate the effect of adding certain concentrations of dandelion to the basal diet on the growth performance, meat quality, blood indexes, and intestinal flora of Hycole rabbits by means of serological detection, ELISA, and 16S rRNA sequencing technologies. This study provides a theoretical reference for exploring the application of traditional Chinese herbal medicines in rabbit breeding, which is of great value in improving rabbit meat quality, reducing the use of antibiotics, and providing healthy meat products for human consumption.

## MATERIALS AND METHODS

### Animals and diets

Dandelion powder (made from dandelion root and stem ground into powder) was purchased from the market of Chinese herbal medicine in Bozhou. Meat rabbit feeding experiments were conducted in rabbit hutches within the Northwest Agriculture and Forestry University Animal Husbandry and Ecological Farm (Yangling, China). In all, 60 weaned Hycole meat rabbits of 35 days of age and similar weight were randomly divided into five groups of 12 rabbits per group, and each meat rabbit was taken to be fed in a single cage. All weaned meat rabbits were pre-fed on normal rations for 3 days with free access to food and water, after which they were fed on different rations according to group. Their body weight was measured weekly, and the remaining rations were weighed to calculate their feed intake. The experiment period was 42 days, and after 42 days, eight rabbits from each group were randomly selected for slaughter. The groups and diets are shown in Table 1. Normal dietary feed ingredients mainly include corn, soybean meal, wheat bran, alfalfa grass meal, stone powder, vitamins, mineral elements, and their complexes, a variety of amino acids, trace elements, and so on. Among them, the guaranteed value of nutrient composition analysis of ordinary diets is shown in Table 2.

**TABLE 1:** Feeding groups and feeding conditions

Group	Diet
Control	Ordinary ration
0.5% dandelion	99.5% Normal ration + 0.5% dandelion
1% dandelion	99% Normal ration + 1% dandelion
1.5% dandelion	98.5% Normal ration + 1.5% dandelion
2% dandelion	98% Normal ration + 2% dandelion

**TABLE 2:** The guaranteed value of nutrient composition analysis of ordinary diets

Ration ingredient	Content (%)
Moisture	≤11%
Crude protein	≥17.0%
Crude fat	≥3%
Crude fiber	≤12%
Crude ash	≤9%
Calcium	1%–1.5%
Total phosphorus	0.5%–0.8%

### Dandelion active ingredient determination

Take 200 mg of dandelion powder sample, add 10 mL of 50% methanol water (methanol: water = 1:1), sonicated for 30 min, take the supernatant for 1 mL in a centrifuge tube at 14,000 rpm for 5 min, remove the supernatant over 0.22 μm pore filter membrane, and put it into the sample bottle for UHPLC-MS/MS (AB Sciex, USA) analysis. Blank samples were treated under the same conditions.

### Sample collection and determination

Determination of daily weight gain: Weighing was performed in the morning before feeding on the 1st, 8th, 15th, 22nd, 29th, 36th, and 43rd days of the trial period. Measurements of feed intake: Daily feed intake was recorded, and feed residuals were weighed before feeding on the 8th, 15th, 22nd, 29th, 36th, and 43rd days of the experimental period. Average feed intake (g/d) = (feed weight (g) − remaining feed weight (g))/7 (d). Measurement of production performance and meat quality: carcass weight is the weight of the rabbit after the removal of skin, head, tail, viscera, carpal joints of the front feet, parts below the hock joints of the hind feet, and blood after slaughter. Carcass rate = carcass weight/pre-slaughter live weight. pH values: 24 h after slaughter, a portable insertable pH meter was inserted into the longest muscle of the back at a depth of 3 mm and averaged over three measurements. Moisture content: 0.5 g of meat was trimmed and placed flat in the center of the total moisture meter tray for direct measurement. Shear force: 24 h after slaughter, the longest muscle of the back to remove fat and connective tissue, from each piece of meat samples in the direction of the muscle fibers to cut the size of 2 cm × 1 cm ×1 cm meat three pieces, each piece of meat samples into a plastic film bag sealed, into the 80°C water bath heating for 30 min, take out the air to cool to the center of room temperature, with a piece of paper to absorb the surface moisture, the use of the tenderness instrument to measure the shear force, the shear force was measured using a tenderness meter, and the cutter was held perpendicular to the meat sample during the measurement, with the maximum force value for the cutting process being the shear force. Meat color: Measure the color of the longest muscle of the back with a portable colorimeter on the surface of the cross-section three times at 24 h after slaughter, and then average the values to get the L* value (brightness), a* value (redness), b* value (yellowness). Cooking rate: the longest muscle of the back was taken 48 h after slaughtering, and the outer membrane and attached fat were stripped off, trimmed into a square cube, weighed about 20 g and placed in a rice cooker with a steam drawer in boiling water for 30 min, and then hung in a cool place for 30 min after steaming and then weighed. Cooking rate (%) = weight of meat sample after steaming/weight of meat sample before steaming × 100. Drip loss (%) = (weight of meat sample before hanging − weight of meat sample after hanging)/weight of meat sample before hanging) × 100. At the end of the 42-day experimental period, all rabbits were fasted for 12 hours, and eight rabbits in each group were randomly selected for venous blood collection, which was stored in blood collection tubes at −20°C for subsequent analysis, and then slaughtered for determining the growth performance and meat quality, and at the same time, the contents of the cecum were collected from the cecum in sterile tubes, which were stored at −80°C for analyzing the microbial composition of the gut. Muscle PH tester testo206-pH2 (Meister, Germany), GR-150 Warner-Bratzler Shear Force Instrument (G-R, USA), Q Exactive Plus Orbitrap, High-resolution instrument (Thermo Fisher, USA), U3000 Ultra-HPLC and autosampler (Thermo Fisher, USA), Ultrasonic cleaner (Beijing Liuyi Instrument Factory, Beijing), and chromatcolumn (Waters, USA).

### Serum parameters assay

The activity of serum diamine oxidase and the concentrations of serum cytokines (interleukin 1b [IL-1β], rabbit interleukin 6 [IL-6], rabbit immunoglobulin G [IgG], rabbit immunoglobulin A [IgA], and rabbit immunoglobulin M [IgM]) were determined using commercial ELISA kits (FANKEW, Shanghai Kexing Trading Co., Ltd, China). Moreover, serum antioxidant-related parameters, including malondialdehyde (MDA), superoxide dismutase (SOD), glutathione peroxidase (GSH-Px), and total antioxidant capacity (T-AOC) were determined using commercial kits (Jiancheng Bioengineering Institute, Nanjing, China) as described previously (23)

### Gut microbiome

Microbial DNA was isolated from cecum contents using a commercial kit, E.Z.N.A Mag-Bind Soil DNA Kit (OMEGA), and the quality of the DNA was checked by electrophoretic analysis before sequencing. DNA concentration was determined using a Qubit 4.0 fluorometer Q33238 (Thermo Fisher, USA). Pyrophosphate sequencing of 16s rDNA was performed on an Illumina HiSeq PE250 platform (Sangon Biotech, Shanghai, China). The library was built using 2 Hieff Robust PCR Master Mix (Yeasen, Shanghai, China) and Hieff NGS DNA Selection Beads (Yeasen, Shanghai, China) kits. We analyzed the classification of 16s rRNA gene sequences (24). Raw sequence data were de-joined, spliced, and quality filtered using CUTADAPT v1.18, PEAR v0.9.8, and PRINSEQ v0.20.4 (25, 26). The remaining high-quality clean labels were then clustered into operational taxonomic units (OTUs) using Usearch v11.0.667 software at 97% sequence similarity (27, 28). The 16s rRNA gene sequence data were deposited in the National Centre for Biotechnology Information (NCBI) Sequence Read Archive (SRA) under the accession number PRJNA1009939.

### Statistical analysis

GraphPad Prism 10.0 statistical analysis software was used for processing, and the data of the measurement information were expressed as mean ± standard error of mean, and one-way ANOVA (one-way ANOVA) was used to compare the means between multiple groups, with other uses of two-by-two comparisons. *P* < 0.05 is considered a statistically significant difference.

## RESULTS

### Analysis of the dandelion active components

The detected compounds were ranked by their mass-spectral response values, and the activity of the top 20 compounds was analyzed as shown in Table 3. The only active ingredient with more than 20% content was rutin (20.169%), while other active ingredients with more than 10% content were quercetin (15.618%), chlorogenic acid (15.321%), and sanguinarine (13.008%) (Table 3). Dandelion powder nutritional composition index was determined to obtain the following: moisture 6.6%; crude protein 15.62%; crude fat 3%; crude fiber 9.3%; and crude ash 21.3%. After adding 2% dandelion powder to the common meat rabbit diet, moisture ≤10.912%; crude protein ≥16.97%; crude fat ≥3%; crude fiber ≤11.95%; crude ash ≤9.24%, which could meet the nutritional requirements of meat rabbits.

**TABLE 3:** Active components and contents of alcohol extract of dandelion top 20 (%）

Rank	Chemical compound	Content (%）	Function
1	Rutin	20.169	Diuretic, antitussive, hypolipidemic, hypotensive, ulcer surface protection, anti-inflammatory, and anti-allergic effects
2	Quercetin	15.618	Hypotensive, anti-inflammatory effects
3	Chlorogenic acid	15.321	Antibacterial, choleretic, hemostatic, leukemic and antiviral, etc.
4	Morin	13.008	Anti-cancer, anti-inflammatory, immunomodulatory, antioxidant
5	Quinic acid	5.802	Enhance bile to lower fat, liver detoxification, prevention of fatty liver
6	Quercetin	5.673	Expectorant, cough suppressant, asthma reliever
7	Neochlorogenic acid	3.429	Antimicrobial effect
8	2-Pyrrolidinecarboxylic acid	1.911	Helicosanoids
9	Scutellarin	1.84	Antioxidant, antitumor, antiviral, and anti-inflammatory activities
10	Citric acid	1.708	Organic acid
11	1,3-Dicafreoylguinic acid	1.282	Antioxidant activity and free radical scavenging activity
12	Cichoric acid	1.135	Anti-inflammatory, enhances immune function and induces cell death
13	α-Linolenic acid	1.128	Fatty acids
14	Narcissoside	0.957	Antioxidant, anti-free radical activity
15	Luteolin	0.846	Anti-inflammatory, antioxidant, and neuroprotective
16	lsochlorogenic acid C	0.822	Antioxidant, anti-inflammatory, antiviral, antifibrotic, inhibition of smooth muscle contraction, hypolipidemic, etc.
17	Sucrose	0.811	Sugar
18	Shikimic acid	0.745	Anti-inflammatory, analgesic
19	Stachydrine	0.651	Sugar
20	Isorhamnetin	0.559	Scavenges oxygen-free radicals, lowers serum cholesterol, protects cardiovascular system, promotes smooth blood flow, etc.

### Effect of adding dandelion on the production performance and meat quality of meat rabbits

**Fig 1:**
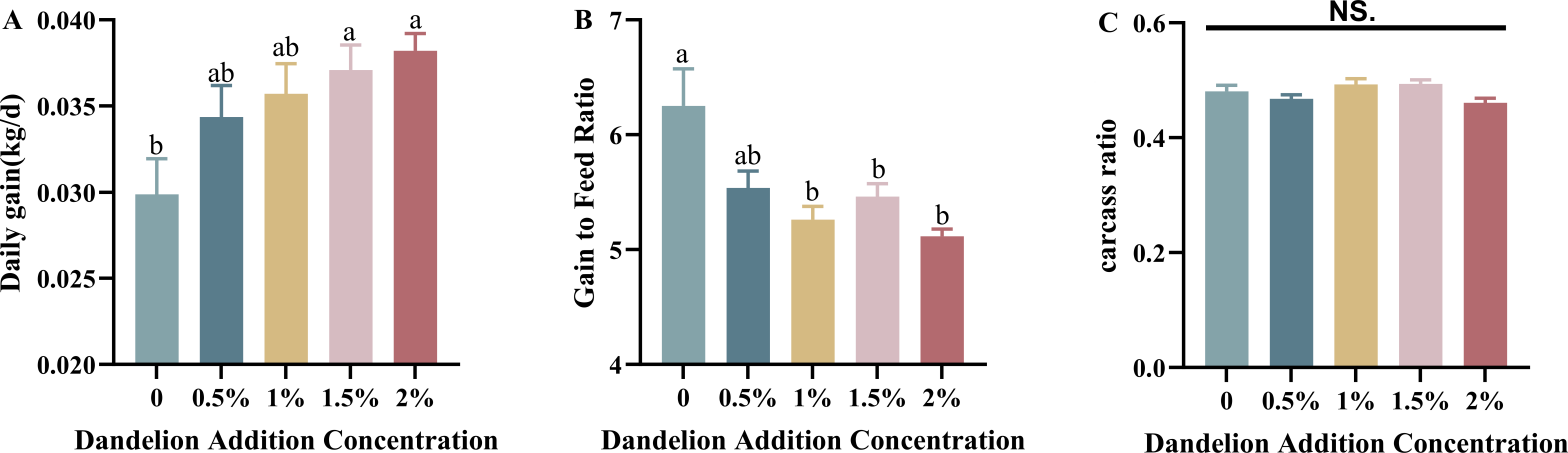
Effects of dandelion supplementation on average daily gain, gain to feed ratio, and carcass ratio of rabbits. Note: Letters indicate significance of differences between treatments at a level of significance of 0.05; errors are expressed as standard errors, same as below.

Statistical analysis of daily weight gain and feed-to-weight ratio of meat rabbits during the experimental period showed that the average daily weight gain of meat rabbits increased gradually with the increase of dandelion concentration. The difference between the 0.5% and 1% dandelion-added groups and the control group was not significant (*P* > 0.05), the difference between the 1.5% dandelion-added group and the control group was significant (*P* < 0.05), and the 2% dandelion-added group had a very significant increase in the daily weight gain compared with that of the control group (*P* < 0.01) (Fig. 1A). The analysis of the feed-to-weight ratio showed that the feed-to-weight ratios of the meat rabbits fed with dandelion were lower than those of the control group, with a significant difference between the 1.5% dandelion-added group and the control group (*P* < 0.05), and the feed-to-weight ratios of the 1% dandelion-added group and the 2% dandelion-added group were significantly lower than those of the control group (*P* < 0.01) (Fig. 1B). The overall tendency of the feed-to-weight ratio decreases with increasing concentration.

**Fig 2:**
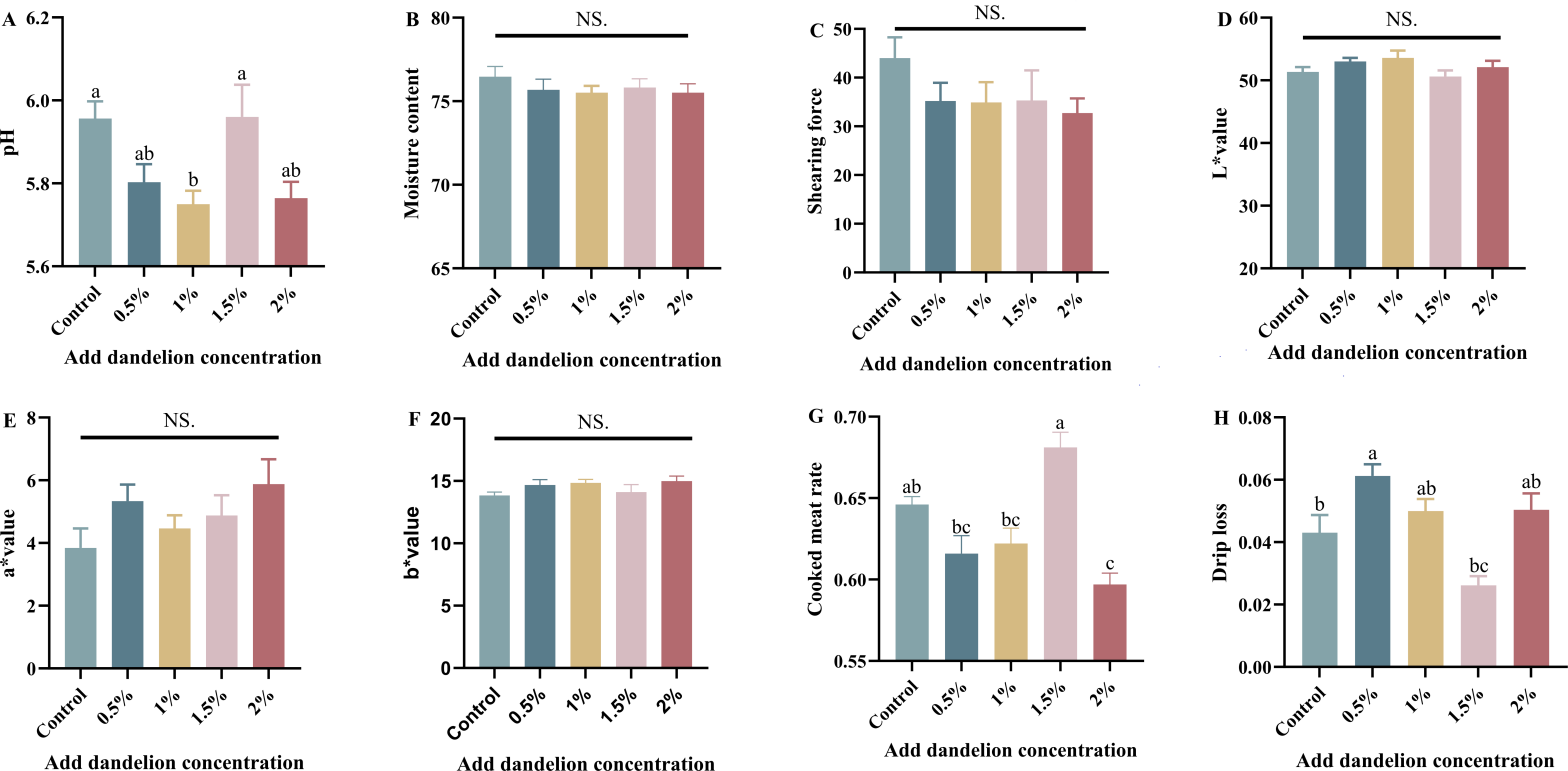
Effects of dandelion feeding on meat quality of domestic rabbit (number of test samples in each group, *n* = 8).

The analysis of production performance showed that the addition of dandelion did not affect the slaughtering rate of meat rabbits, and the slaughtering rate of the 1% and 1.5% dandelion groups was slightly higher than that of the other experimental groups and the control group, and the differences between the four experimental groups and the control group were not significant (*P* > 0.05) (Fig. 1C). The meat quality of meat rabbits was further analyzed, as shown in Fig. 2, by statistically analyzing the differences between the addition of different gradient concentrations of dandelion feeding on the meat quality of meat rabbits and the control group by the items of pH, water content, shear force, meat color, cooked meat rate, and drip loss. The pH values of the muscles in the control and experimental groups were between 7.1 and 7.2 before slaughter. After slaughter, the pH values decreased due to the production of lactic acid and phosphoric acid from the decomposition of myo-glycogen, but both of them were within the normal range (29), and the pH values of the 1% dandelion group differed significantly from those of the control group (*P* < 0.05), while the differences between the dorsal longest muscle of the rest of the experimental groups and those of the control group were insignificant (*P* > 0.05) (Fig. 2A). The cooked meat rate was slightly lower in the groups with 0.5% and 1% dandelion and slightly higher in the group with 1.5% dandelion, neither of which resulted in significant differences (*P >* 0.05), while the 2% dandelion-added group was significantly lower than that of the control group (*P* < 0.05) (Figure G). The meat samples were suspended by silk thread for drip test, and it was found that the drip loss of the 0.5% dandelion-added group was significantly higher than that of the control group (*P* < 0.05), the drip loss of the 1.5% dandelion-added group was significantly lower than that of the control group (*P* < 0.05), and the difference between the remaining two groups and the control group was not significant (*P* > 0.05) (Fig. 2H). Water content and shear were slightly lower in all four experimental groups than in the control group, but the differences were not significant (*P* > 0.05) (Fig. 2B and C), and the L*, a*, and b* values of meat color in the four experimental groups were not significantly different from the control group (*P* > 0.05) (Fig. 2D through F).

### Effect of dietary dandelion supplementation on meat rabbit blood routine

To evaluate the effect of adding dandelion to the feed on the physiological and biochemical indices of rabbit blood, we conducted a routine blood test on the rabbit blood. Adding different concentrations of dandelion powder to the diet of meat rabbits had a significant effect on the blood biochemistry of meat rabbits in terms of the number of white blood cells (WBC), the number of neutrophils (Neu#), the number of lymphocytes (Lym#), the number of eosinophils (Eos#), the number of basophilic granulocytes (Bas#), the percentage of neutrophils (Neu%), the percentage of lymphocytes (Lym%), the number of eosinophils (Eos#), percentage of basophilic granulocytes (Bas%), number of red blood cells (RBCs), hemoglobin (HGB), erythrocyte cumulative pressure (HCT), mean corpuscular volume of red blood cells (MCV), mean corpuscular hemoglobin content of red blood cells (MCH), mean corpuscular hemoglobin concentration of red blood cells (MCHC), red blood cell distribution width coefficient of variation (RDW-CV), red blood cell distribution width standard deviation (RDW-SD), platelet number (PLT), mean platelet volume (MPV), platelet distribution width (PDW), and plateletocrit (PCT) were not significantly affected, but some of these indicators were different in different groups, with different degrees of exceeding the range of normal values, as shown in Table 4.

**TABLE 4:** Blood routine test

Items (unit)	0	0.50%	1%	1.50%	2%	Range
White blood cell count (10^9^ /L）	8.28 ± 1.980	9.102 ± 1.697	8.72 ± 1.639	7.156 ± 0.808	7.994 ± 1.753	3.00-13.50
Number of neutrophils (10^9^ /L）	2.384 ± 0.801	2.918 ± 0.456	2.198 ± 0.947	1.654 ± 0.264	1.852 ± 0.946	0.50-6.60
Number of lymphocytes (10^9^ /L）	3.648 ± 1.335	3.62 ± 1.443	4.31 ± 0.821	4.104 ± 0.993	4.396 ± 1.240	1.00-6.80
Number of monocytes (10^9^ /L）	1.368 ± 0.274	1.636 ± 0.315	1.536 ± 0.549	0.878 ± 0.176	0.916 ± 0.309	0.08-1.51
Number of eosinophils (10^9^ /L）	0.826 ± 0.139	0.868 ± 0.237	0.612 ± 0.251	0.448 ± 0.136	0.706 ± 0.336	0.00-0.51
Number of basophils (10^9^ /L）	0.054 ± 0.020	0.06 ± 0.036	0.064 ± 0.019	0.072 ± 0.023	0.124 ± 0.064	0.00-0.76
Percentage of neutrophils (%）	29.04 ± 6.418	32.6 ± 5.560	24.38 ± 7.003	23.24 ± 4.143	22.58 ± 8.347	14.0-62.0
Percentage of lymphocytes (%）	42.74 ± 7.964	38.42 ± 13.177	50.68 ± 12.568	56.78 ± 7.458	55.9 ± 13.726	25.0-82.0
Percentage of monocytes (%）	16.76 ± 1.432	18.44 ± 4.698	17.3 ± 3.926	12.5 ± 2.904	11.32 ± 2.251	2.0-15.0
Percentage of eosinophils (%）	10.76 ± 3.844	9.86 ± 3.466	6.88 ± 2.624	6.46 ± 2.550	8.68 ± 3.485	0.0-6.0
Percentage of basophils (%）	0.7 ± 0.253	0.68 ± 0.248	0.76 ± 0.195	1.02 ± 0.311	1.52 ± 0.487	0.0-8.0
Number of red blood cells (10^12^ /L）	5.72 ± 0.637	5.892 ± 0.427	6.138 ± 0.581	6.33 ± 0.880	6.102 ± 0.624	3.40-6.50
Hemoglobin (g/L）	118.2 ± 17.371	122.2 ± 7.332	129.6 ± 7.301	134.6 ± 8.678	135.4 ± 10.407	80-140
Erythrocyte accumulation (%）	39.76 ± 5.579	41.68 ± 2.746	44.2 ± 3.120	45.3 ± 3.121	45.34 ± 3.340	25.0-42.0
Mean red blood cell volume (fL）	69.42 ± 5.150	70.88 ± 2.878	72.2 ± 3.821	72.1 ± 5.221	74.56 ± 2.936	60.0-80.0
Mean red cell hemoglobin content (pg）	20.62 ± 1.454	20.78 ± 0.813	21.18 ± 1.431	21.48 ± 1.660	22.26 ± 0.754	19.0-25.0
Mean erythrocyte hemoglobin concentration (g/L）	296.8 ± 5.036	293.2 ± 6.431	293.4 ± 6.025	297.6 ± 3.209	298.2 ± 2.950	300-360
Coefficient of variation of erythrocyte distribution width (g/L）	17.58 ± 4.640	16.68 ± 3.842	14.5 ± 1.602	14.2 ± 1.145	15.32 ± 2.041	10.5-22.0
Standard deviation of erythrocyte distribution width (fL）	41.06 ± 7.255	39.78 ± 8.039	36.08 ± 3.555	35.22 ± 2.208	39.64 ± 4.860	28.0-65.0
Number of platelets (10^9^ /L）	510.8 ± 142.272	281.4 ± 152.533	373.6 ± 192.708	337.4 ± 109.926	283.8 ± 163.663	100-1250
Mean platelet volume (fL）	8.1 ± 0.525	7.98 ± 0.700	8.04 ± 0.451	7.76 ± 0.410	7.86 ± 0.416	4.0-7.8
Platelet distribution width	16.02 ± 0.371	16.4 ± 0.762	16.26 ± 0.397	16.26 ± 0.195	16.62 ± 0.722	12.0-17.5
Plateletocrit (%）	0.4074 ± 0.101	0.2300 ± 0.127	0.3050 ± 0.160	0.2608 ± 0.082	0.2214 ± 0.127	0.0300-0.790

**Fig 3:**
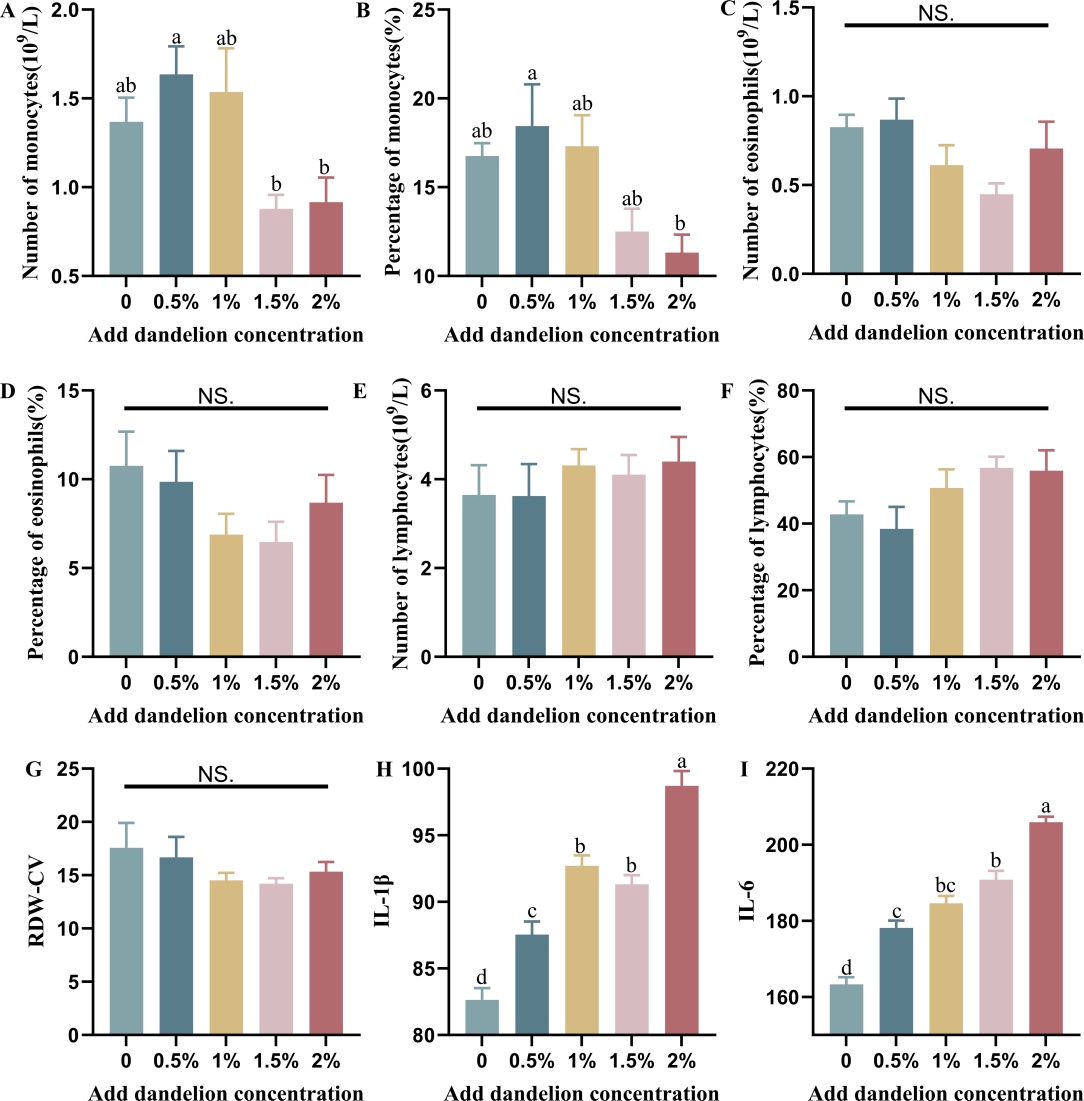
Effect of dandelion on serum immune indices of rabbits (number of test samples in each group, *n* = 8).

Blood biochemical indicators are markers of animal health and internal metabolic status (30). To display the differences in the main blood indicators among different groups more clearly, we have visualized them. As shown in Fig. 3A, the number of monocytes in the 0.5% and 1% dandelion-added groups was higher than that in the control group, but the difference was not significant (*P* > 0.05). Meanwhile, the number of monocytes in the 1.5% and 2% dandelion-added groups was significantly lower than that in the 0.5% dandelion-added group (*P* < 0.05), and also lower than that of the control group, but the difference was not significant (*P* > 0.05). The percentage of monocytes in the 0.5% dandelion-added group was slightly higher than in the control group, but gradually decreased as the amount of dandelion added to the diet increased. The percentages of monocytes in the 1.5% and 2% dandelion-added groups were lower than that of the control group (*P* > 0.05), and the percentage of monocytes in the 2% dandelion-added group was significantly lower than that of the 0.5% dandelion-added group (*P* < 0.05) (Fig. 3B). Although the number and percentage of eosinophils did not differ significantly among the groups, a decreasing trend could be observed in the 1% and 1.5% dandelion-added groups (*P* > 0.05) (Fig. 3C and D). Lymphocytes were increased in the experimental group with 1%, 1.5%, and 2% dandelion-added compared to the control group and the group with 0.5% dandelion-added (*P* > 0.05) (Fig. 3E and F). The coefficient of variation of erythrocyte distribution width, although not significant among the groups, but also showed a decreasing trend with the increase in dandelion (*P* > 0.05) (Fig. 3G). IL is a cytokine produced by a variety of cells and can act on a variety of cells and plays an essential role in transmitting information, regulating and activating immune cells, and mediating the proliferation and differentiation of lymphoid B cells and lymphoid T cells.

### Effect of the diet supplementation of dandelion on the immune performance of meat rabbits

To evaluate the effect of adding dandelion to the feed on the immune performance of rabbits, we used a commercial kit to detect immune-related indicators in the serum. As shown in Fig. 3H, the serum IL-1β in the experimental group with different concentrations of dandelion powder added to the diet of meat rabbits was significantly higher than that in the control group (*P* < 0.01), except for a slight decrease in the group with 1.5% dandelion powder added to the diet, and the overall trend was still higher with the increase in dandelion powder content. As shown in Fig. 3I, similar to the results of IL-1β, the serum IL-6 levels in the rabbit diets with dandelion powder were also significantly higher than those in the control group (*P* < 0.01), and the differences between the 1% dandelion-added group and the 0.5% and 1.5% dandelion-added groups were not significant. Overall, however, serum IL-6 levels increased with dandelion powder.

**Fig 4:**
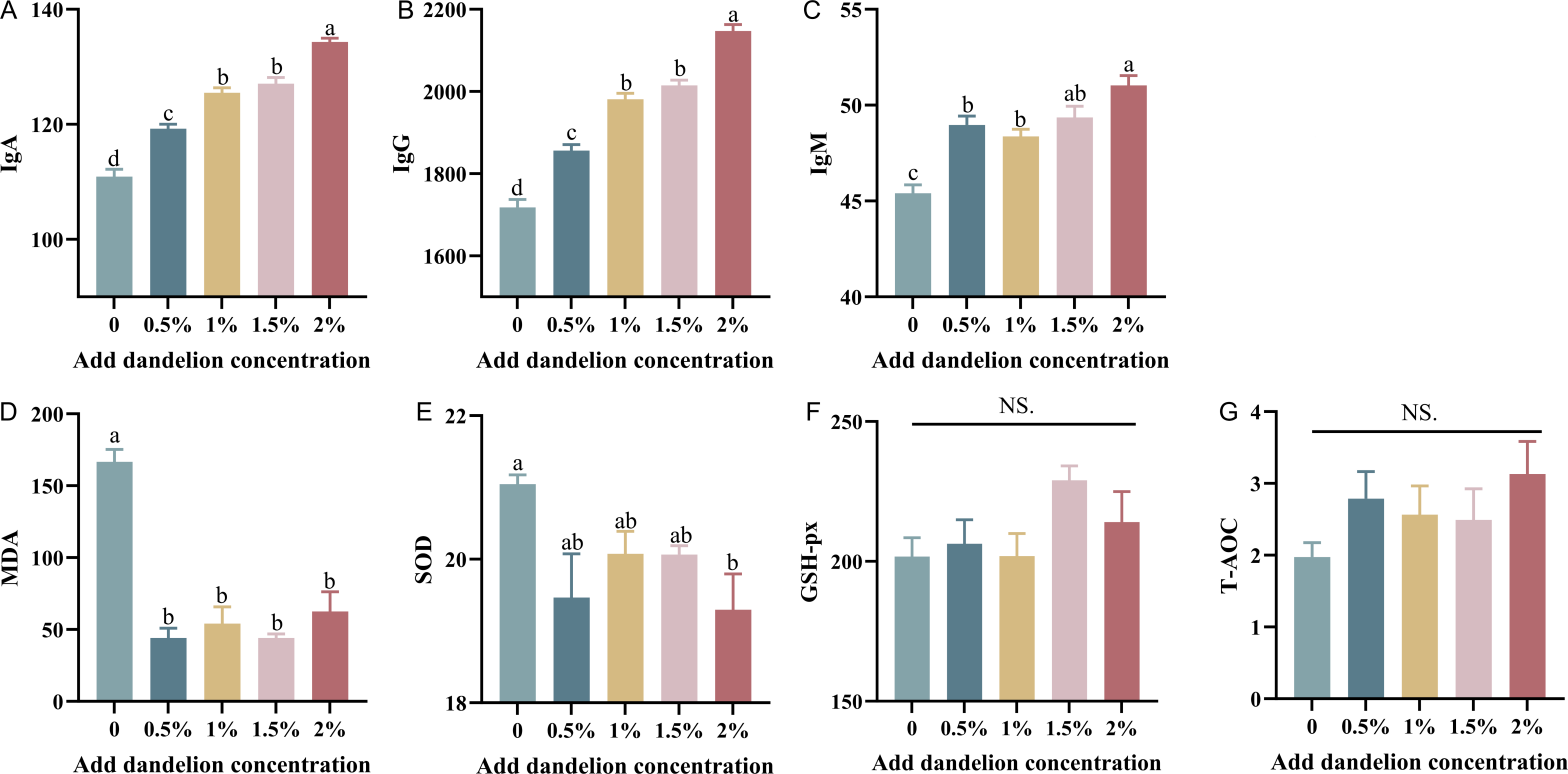
Effect of dandelion on serum immunoglobulin and antioxidant capacity in rabbits (number of test samples in each group, *n* = 5).

The changes in immunoglobulin content in the serum of meat rabbits by dandelion addition were tested by an ELISA kit, as shown in Fig. 4. It is worth noting that the trends of IgA and IgG changes were consistent, and the IgA and IgG contents of the experimental groups with different concentrations of dandelion powder added to the diets of meat rabbits were significantly higher than that of the control group (*P* < 0. 01). Specifically, the IgA and IgG content of the 1% and 1.5% dandelion-added groups were both IgA and IgG contents of the 1% and 1.5% dandelion-added group were significantly higher than those of the 0.5% dandelion-added group (*P* < 0.01). The IgA and IgG contents of the 1.5% dandelion-added group were higher than those of the 1% dandelion-added group, but the difference was not significant. The contents of the 2% dandelion-added group were extremely higher than those of the other experimental groups (*P* < 0.01), and in general, the contents of IgA and IgG were higher with the increase in the amount of dandelion added to the diets. As shown in Fig. 4C, the serum IgM content of each experimental group of meat rabbits with dandelion powder added to the diet was significantly higher than that of the control group (*P* < 0.01). The differences between the 0.5%, 1%, and 1.5% dandelion-added groups were not significant, and the differences between the 1.5% and 2% dandelion-added groups were not significant. The IgM content of serum in the 2% dandelion-added group was significantly higher than that of the 1% dandelion-added group (*P* < 0.01), and significantly higher than that of the 0% dandelion-added group (*P* < 0.01), and significantly higher than that in the 0.5% dandelion-added group (*P* < 0.05).

### Effect of dietary dandelion supplementation on antioxidant properties in meat rabbits

At the end of the experimental period, five individuals in each group were randomly selected for the antioxidant indexes of meat rabbits, and the antioxidant performance indexes included MDA, GSH-PX, SOD, and T-AOC, which were determined by the kit from Nanjing Jianjian Institute of Biological Engineering, etc. The MDA was measured by the thiobarbituric method, and it can be seen that the MDA values of 0%, 1%, 1%, and 2% dandelion-added groups were greatly lower than the control group (*P* < 0.05) (Fig. 4D). In the determination of SOD in the serum of meat rabbits using the hydroxylamine method, it was found that the 2% dandelion-added group was significantly lower than that of the control group (*P* < 0.05), and the remaining three groups were not significantly different from that of the control group (*P* > 0.05) (Fig. 4E). The thiobarbituric acid method for the determination of GSH-px showed that the serum GSH-px content of the dandelion-added group was higher than that of the control group, but there was no significant difference (*P* > 0.05) (Fig. 4F). Colorimetric determination of T-AOC content in the serum of meat rabbits, as shown in Fig. 4G, showed that the T-AOC in 0.5%, 1%, 1.5%, and 2% dandelion-added groups was higher than that in the control group, but the difference was not significant (*P* > 0.05).

### Effect of dandelion content on the diversity of alpha flora in the gut of meat rabbits

Five diversity indices were selected to summarize the alpha diversity of the gut flora of the hare, as shown in Table 5. The Shannon index showed that the flora diversity of the 1% and 1.5% treatment groups was significantly lower than that of the control group (*P* < 0.05), and that of the 0.5% and 2% groups was also lower than that of the control group, but was not significant (*P* < 0.05). The results of the Simpson index of the diversity of the intestinal flora also showed a similar result to that presented by the Shannon index presented similar results, the values of 1% and 1.5% groups were significantly higher than the control group (*P* < 0.05), indicating that these two groups were significantly less diverse than the control group. From the Chao1 index, the 0.5% dandelion addition group had the highest abundance with the highest number of bacterial species and was significantly higher than the other groups (*P* < 0.05), ranked from high to low 0.5% > 2% > 0%> 1.5% > 1%. The results of the Ace index showed similar results, with the difference being that the Ace index did not differ significantly between the treatment groups. It can be seen that there is no simple linear relationship between the level of dandelion content in the feed and the abundance of gut flora in the cecum of the meat rabbit, but the treatment group with 1% dandelion content was the least rich in bacterial species. In terms of homogeneity, the degree of homogeneity was, in descending order, 0% > 2% > 0.5%> 1.5% > 1%.

**TABLE 5:** Effects of dandelion supplementation on alpha diversity of cecal intestinal flora in rabbits^*a*^

Groups	Shannon index	Chao1 index	Ace index	Simpson index	Shannon index
0%	4.48 ± 0.33^a^	663.77 ± 28.14^ab^	656.79 ± 19.51	0.04 ± 0.03^c^	0.70 ± 0.05^a^
0.50%	4.18 ± 0.12^ab^	699.55 ± 27.34^a^	682.74 ± 14.39	0.07 ± 0.01b^c^	0.65 ± 0.02^ab^
1.00%	3.17 ± 0.53^c^	624.58 ± 24.64^b^	618.09 ± 43.54	0.14 ± 0.04^a^	0.51 ± 0.07^c^
1.50%	3.48 ± 0.37^bc^	628.92 ± 58.20^b^	624.94 ± 62.03	0.12 ± 0.04^ab^	0.55 ± 0.05^bc^
2.00%	4.33 ± 0.45^a^	679.41 ± 27.53^ab^	675.32 ± 24.34	0.05 ± 0.03^c^	0.67 ± 0.07^a^

^*a*^ Note: Letters indicate the significance of differences between treatments. Different letters denote a significant level of 0.05; errors are expressed as the standard error of the mean.

### Beta diversity analysis of intestinal microflora in meat rabbits supplemented with dandelion content

**Fig 5:**
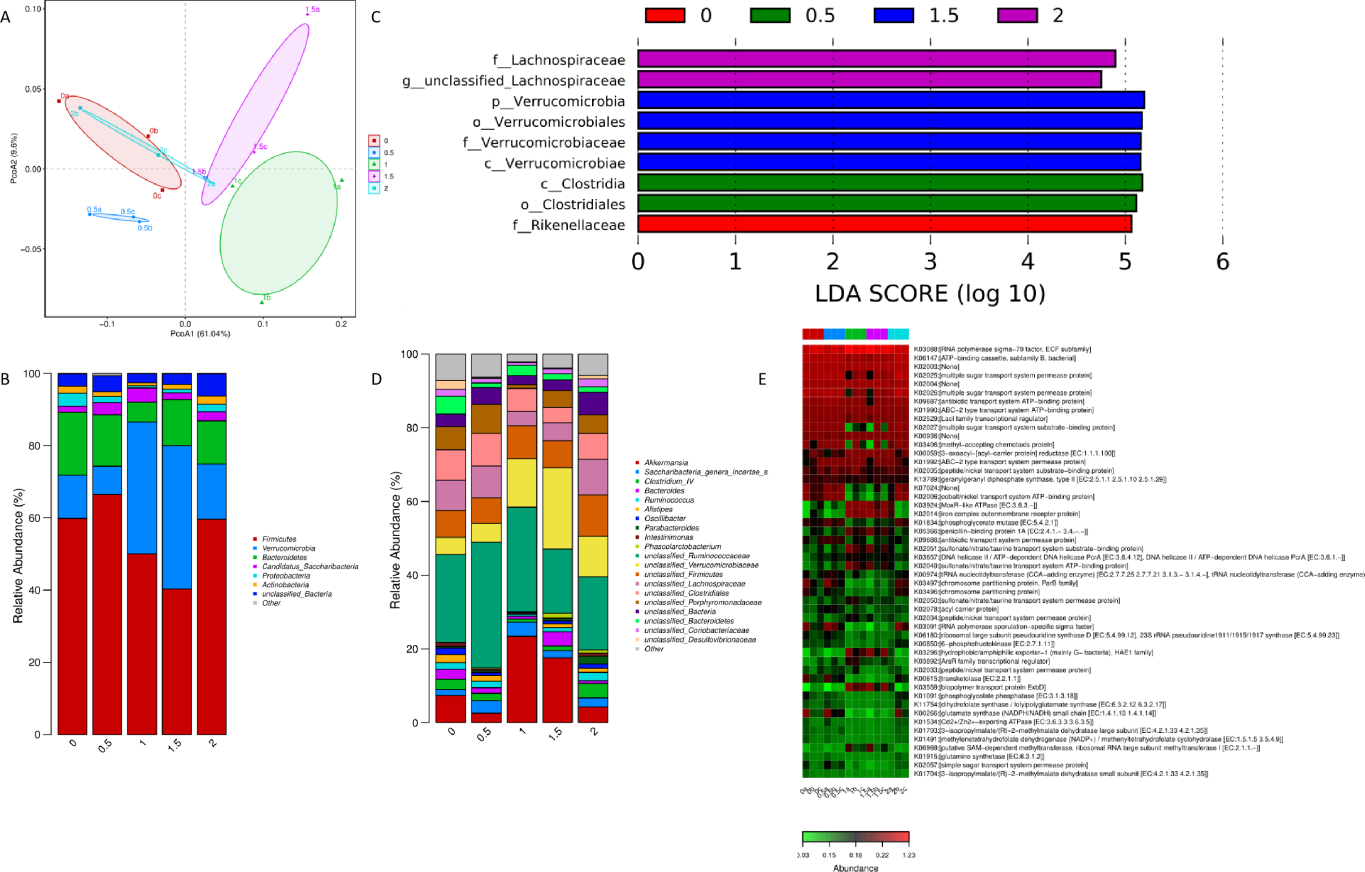
The effect of dandelion on the intestinal flora of meat rabbits (number of test samples in each group, *n* = 3). (A) PCoA analysis based on Weighted Unifrac algorithm; (B) Average structural differences among treatment groups at phylum level; (C) LEFSef analysis of each processing group; (D) Average structural difference of treatment groups at genus level; (E) KEGG functional heat map.

Figure 5A showed that principal component 1 accounts for 61.04% of the variation in the community, and principal component 2 accounts for 9.6% of the variation in the community, for a total of 70.64% of the variation. Among them, the communities between different treatment groups can be roughly separated from each other, but the 2% treatment group has overlapping positions with the control group and the 1.5% treatment group, in addition, the three samples within the 0.5% treatment group are relatively closest to each other and have smaller differences, while the other treatment groups have larger differences in the communities between the samples.

### Structural differences at the level of the cecum of meat rabbits

As can be seen in Fig. 5B, in the control group, the horizontal composition of cecal intestinal flora phyla of meat rabbits was as follows: Firmicutes (59.85%), Verrucomicrobia (12.00%), Bacteroidetes (17.39%), Candidatus Saccharibacteria 1.63%, Proteobacteria 3.62%, Actinobacteria 1.96%, Unclassified Bacteria 3.38%, and others 0.16%. Thick-walled Bacteria, Actinobacteria, and Warty Microbacteria were the dominant phyla in the control group. Among the groups with dandelion addition, the composition of phylum levels in the 2% addition group was similar to that of the control group, whereas the 0.5%, 1%, and 1.5% groups all differed considerably from that of the control group. There is some evidence that the percentage of thick-walled phyla in the 0.5% group increased to 66.53%, and the relative percentages of anamorphic phyla and warty microflora decreased to 14.27% and 7.78%, respectively. The percentage of thick-walled phyla in the 1% group fell to 50.05%, and the percentage of warty microflora increased dramatically to 0.16%. Micromycetes increased substantially to 36.55% and *Mycobacterium falciparum* declined to 5.53%. In the 1.5% group, the percentage of thick-walled Mycobacterium enabled fell to only 40.27%, *Mycobacterium verrucosum* to 39.69%, and Mycobacterium anisopliae to 12.77%.

### Structural differences at the genus level of meat rabbits

As shown in Fig. 5D, roughly 20 genera are found in the cecum of the meat rabbit in proportions greater than 0.05%. In the control group, the genus with the largest percentage was a genus of the family Ruminococcaceae (Unclassified Ruminococcaceae) with 23.87%, followed by a genus of the order Clostridiales (Unclassified Clostridiales) with 8.34%, and other genera with larger percentages were Akkermansia with 7.39%, Unclassified Firmicutes with 7.24%, Unclassified Porphyromonadaceae with 6.25%, Unclassified Lachnospiraceae with 6.25%, and Unclassified Lachnospiraceae with 6.25%. Lachnospiraceae at 8.23%, and a genus of Unclassified Verrucomicrobiaceae at 4.61%. The percentage of a genus of Unclassified Ruminococcaceae (Unclassified Ruminococcaceae) was fluctuating with the addition of dandelion, with the highest percentage of 34.04% in the 0.5% addition group and the lowest percentage of 17.42% in the 1.5% addition group. The percentage of unclassified Verrucomicrobiaceae genera had a tendency to increase and then decrease, rising to a maximum of 22.06% in the 1.5% additive group. The percentage of Akkermansia also fluctuated with the addition of dandelion and was significantly higher than that of the control group in the 1% addition group and the 1.5% addition group, with 23.43% and 17.63%, while it was rather not as high as in the control group in the 0.5% added and 2% added groups.

### Discriminant analysis of LEfSe differential species in meat rabbits supplemented with dandelion content

LEfSe (Linear discriminant analysis Effect Size, Linear Discriminant Analysis and Influence Factor) is used to discover the genetic or functional features that best explain differences between groups in two or more samples from different biological conditions or environments, and the extent to which these features contribute to the differences between groups. Using an LDA Score (log10) of 4.0 or higher as the discriminating criterion, there were nine OTUs that contributed significantly to the differences in colony structure between groups, as shown in Fig. 5C. In the control group, *Rikenellaceae* had a greater influence. In the 0.5% addition group, it was *Clostridiales* and *Clostridia* that had a greater influence, and in the 1.5% addition group, it was *Verrucomicrobia*, *Verrucomicrobiae*, *Verrucomicrobiales,* and *Verrucomicrobiaceae* that had more implications. Within the 2% additive group, it is the *Unclassified Lachnospiraceae* that are more influential.

### Analysis of PICRUSt function predictions for gut microflora in meat rabbits fed a diet supplemented with dandelion content

All cecum gut flora of hares were annotated in the KEGG database in terms of function and corresponding abundance, and the top 50 most abundant functions were selected and plotted in a heatmap, the results of which are shown in Fig. 5E. As can be seen, the functional abundance values were responsive to the amount of dandelion added, and it was more obvious that there was a significant difference in the functional abundance between the 1% added group and the 1.5% added group and the other treatment groups (0%, 0.5%, and 2%), and the functions with significantly lower abundance were K02025 and K02026: polysaccharide transporter system permease proteins, K02027: polysaccharide transporter system substrate-binding proteins, K03406: methyl receptor chemotactic protein, K02006: cobalt/nickel transport system ATP-binding protein, K01834: phosphoglycerol translocase, K00974: tRNA nucleotidyltransferase (Cca-adductase), K03497: chromosome segmentation protein, PARB family, K03496: chromosome segmentation proteins, K00266: glutamate synthase (NADPH/NADH) small chain, K02057: simple sugar transport system permease proteins, and others. Functions with a significant increase in abundance include K00059: 3-oxoacyl-acyl carrier protein, K02014: iron complex outer membrane receptor protein, K03924: MoxR-like ATPase, and K05366: penicillin-binding protein 1A, K02051: Sulfonate/nitrate/taurine transport system substrate-binding protein, K02049: Sulfonate/nitrate/taurine transport system ATP-binding protein, K02050: Sulfonate/nitrate/taurine transport system permease protein, K02034: Polypeptide/nickel transport system permeability enzyme protein, K03559: biopolymer transporter protein ExbD, K06969: putative SAMRNA-dependent methyltransferase, ribosomal-big-subunit methyltransferase I, and others.

## DISCUSSION

Previous studies have shown that the addition of herbs to feed significantly enhances immunocompetence (31). Dandelion, a perennial herb, is high in sugar and protein and has a high medicinal value. Some studies have shown that dandelion may have potential hypolipidemic and antioxidant effects (9) and inhibit viral polymerase activity and viral nucleoprotein levels (10), as well as some antimicrobial and antiparasitic effects (32). The aim of this study was to assess the effects of adding a moderate amount of dandelion meal to the diet on growth performance, meat quality, immunocompetence, and gut flora of weaned meat rabbits.

In this study, to verify the effect of dandelion on the feeding of meat rabbits, four experimental groups were set up to add different amounts of dandelion to the diet and to analyze its effect on production performance and meat quality by comparison with the control group. The test results showed that the addition of dandelion powder to the diet significantly increased the daily weight gain of meat rabbits and significantly decreased the feed-to-weight ratio (*P* < 0.05). This is consistent with the previous research findings on the components of dandelion in other animals (33, 34). It was found that there was some degree of diarrhea in the control and low-dose dandelion supplement groups and no diarrhea in the high-dose dandelion supplement group, which may have contributed to the higher daily weight gain and lower feed-to-weight ratio in the dandelion supplement group. Previous studies have shown that the components of dandelion can significantly reduce the diarrhea rate in broilers (33). The phenomenon of reduced diarrhea has further piqued our interest. Could this be related to the antioxidant and anti-inflammatory properties of dandelion? Therefore, in subsequent experiments, we focused on the changes in the physiological and biochemical indicators, immune performance, and antioxidant capacity of rabbits.

Through the slaughtering experiment, it was found that the addition of dandelion to the diets of meat rabbits did not affect the muscle moisture content, shear force, and meat color, and although there were some differences in the pH values after slaughtering, they were all within the normal range. Further analysis of meat quality showed that the differences in slaughtering rate between groups were not significant, with the 1% and 1.5% dandelion-added groups being slightly higher than the other experimental groups and the control group, and the increase in slaughtering rate also helped to improve the economic benefits of the farmers. The cooked meat rate of the 0.5% and 1% dandelion-added groups was lower than that of the control group and reached a peak in the 1.5% dandelion-added group, where it was greater than the control group, but the difference was not significant (*P* > 0.05). The rate of cooked meat was greater than that of the control group but not significant (*P* > 0.05), followed by a decreasing trend in the 2% dandelion-added group, and a similar phenomenon was observed in the drip loss. As a result, dandelion can enhance the performance of meat rabbits to a certain extent without compromising the quality of the meat.

The antioxidant capacity of an animal organism, in response to its ability to scavenge free radicals, provides a dynamic and balanced tissue environment that includes both enzymatic and non-enzymatic systems. Dandelion can play the role of a nutrient and active ingredient in the growth process of meat rabbits, which, in turn, promotes the function of the antioxidant system (35). Blood components are the material basis of metabolism, and changes in their content can reflect the metabolic function and physiological activity patterns of the animal organism. Blood routine indicators can reflect the health status or disease state of the animal, and plasma biochemical indicators are commonly used to reflect the changes in the metabolism of nutrients and tissue and organ function of the animal organism (36, 37). In this experiment, we first tested the blood routine indexes of meat rabbits and found that all the indexes did not significantly exceed the normal range (*P* > 0.05), but some of the indexes had different degrees of elevation, which indicated that the addition of an appropriate amount of dandelion had a certain effect on the blood components of meat rabbits, but it was not significant. Neutrophils, monocytes, and macrophages secrete pro-inflammatory cytokines, such as TNF, IFN, IL-16, and IL-1β, after recognizing microorganisms such as bacteria and fungi. These cytokines activate neutrophils and macrophages, leading them to phagocytose invading pathogens. The number of mononuclear cells in the 0.5% dandelion-added group was slightly higher than that in the control group and 1% dandelion-added group, and the number of mononuclear cells in the 1.5% and 2% dandelion-added groups was significantly lower than that in the 0.5% dandelion-added group. This phenomenon may be related to the low temperature of rabbit hutches, the weak ability of weaned rabbits to keep themselves warm, and the diarrhea of varying degrees in some of the litters in the control group, the 0.5% and 1% dandelion-added groups, and low immunity of meat rabbits. In the control, 0.5% and 1% dandelion-added groups, some rabbits had diarrhea of varying degrees. In this experiment, the number and percentage of eosinophils in the 1% and 1.5% dandelion-added groups were lower than in the other experimental groups and the control group. Lymphocytes increased with the concentration of dandelion powder added to the diet, possibly due to the fact that dandelion enhanced the proliferation of lymphocytes, which, in turn, improved the immune function of the meat rabbits, enhancing their ability to resist external viruses. In addition, the coefficient of variation in the distribution of erythropoiesis was higher in the control and 0.5% dandelion-added groups compared to the rest of the experimental group, possibly due to nutritional anemia caused by diarrhea in the meat rabbits.

Serum MDA level can reflect the degree of lipid peroxidation reaction and brain damage. SOD and GSH-PX could effectively inhibit the effect of oxygen radicals on the brain cells and protect the neurovascular function. GSH-PX could effectively inhibit the damage of oxygen-free radicals to brain cells and has the effect of protecting neurovascular (38). It has been shown that dandelion polysaccharides significantly increased GSH-PX and T-AOC in serum and liver tissues of mice (*P* < 0.05) and significantly decreased MDA content (*P* < 0.05) compared to the control group after adding dandelion to mouse diets (39). Similar findings are obtained in the present study. The serum MDA of the test group was significantly lower than that of the control group, and the addition of dandelion powder to the diet reduced the degree of lipid peroxidation in meat rabbits, and dandelion powder played a role in enhancing the antioxidant capacity of meat rabbits to some extent. However, the addition of dandelion powder to the diet did not improve the serum SOD content of rabbits. When the concentration of dandelion powder was increased to 2%, the serum SOD content even decreased significantly, which indicated that dandelion powder did not improve the antioxidant capacity of meat rabbits by increasing the SOD level. The significant reduction in the 2% group may possibly be due to the influence of other factors, such as environmental and temperature factors. In this experiment, the serum GSH-px content of the meat rabbits did not differ significantly between the groups, but as the concentration of dandelion powder in the diet increased, the serum GSH-px content of the meat rabbits increased more significantly at 1.5% dandelion powder addition. Previously, in an experiment in which dandelion powder was added to a high cholesterol diet fed to rabbits, it was found that hepatic GSH-px was significantly higher in the experimental group supplemented with dandelion powder than in the positive control group, whereas there was no significant difference from the negative control group fed a normal diet (9), suggesting that the effect of dandelion supplementation on the content of GSH-px may also be related to the cholesterol content of the diet. T-AOC reflects the total antioxidant level composed of antioxidant substances such as vitamin C and vitamin E, and antioxidant enzymes such as superoxide dismutase and glutathione peroxidase in the organism (40). In this study, although the total antioxidant capacity of the experimental group with the addition of dandelion powder to the ration was not significantly different from that of the control group (*P* > 0.05), the dandelion-added group showed an elevated trend compared with the control group, indicating that the addition of dandelion powder can improve the antioxidant capacity of meat rabbits to a certain degree, and our results were similar to those of previous studies (7, 41).

Furthermore, we examined rabbit serum for the immune indicators IgA, IgM, and IgG. Serum immunoglobulin levels are commonly used as a measure of the body’s humoral immunity (42). IgG is the leading component of immunoglobulins in serum, accounting for approximately 75% of all immunoglobulins in serum, and therefore plays an important role in antibacterial and antiviral agents as well as immunomodulation (43). IgA is second only to IgG in serum and is divided into serum and mucosal types. Serum IgA accounts for about 10%–20% of all immunoglobulins in serum (44). IgM accounts for approximately 5%–10% of all immunoglobulins in serum and is the largest molecular weight class of immunoglobulins. IgM is the largest molecular weight class of immunoglobulins and exists as a pentamer. IgM is a highly effective antibody due to its ability to activate complement and its special pentameric structure, which makes it much more effective than IgG (45). In this experiment, IgA and IgG had the same rising trend, and the addition of different concentrations of dandelion significantly increased the serum levels of IgA and IgG, and the levels increased with the concentration of dandelion powder. The addition of dandelion also significantly increased the serum levels of IgM, and the levels of IgM were significantly higher in all four groups compared with the control group, and the levels of IgM were significantly higher in the group of 2% dandelion. The IgM content was also significantly higher in the 2% dandelion-added group than in the 0.5% and 1% dandelion-added groups.

The gut microbiota is involved in nutrient metabolism, the development of innate immunity, and the clearance of pathogens (46, 47). Rabbits are typical herbivorous animals. They rely on gut microbiota (especially cecal microbiota) to decompose cellulose and produce short-chain fatty acids, which are absorbed by cecal epithelial cells, metabolized into energy in the liver, and help maintain the intestinal pH value (48). Intestinal microbiota can also regulate the host immune response through the “microbiota-immune axis” to maintain immune homeostasis. Commensal bacteria activate dendritic cells, promote the differentiation of regulatory T cells, and inhibit excessive inflammation (49, 50). In this experiment, meat rabbits were fed with dandelion in varying doses, and their cecal gut flora was sequenced. The results at the portal level show a decrease in the percentage of Firmicutes and an increase in the percentage of Verrucomicrobia in the cecum of meat rabbits supplemented with 1% and 1.5% of dandelion. Previous studies have shown a significant association between elevated Firmicutes content and weight gain in humans (51), which is not consistent with the results in this study. It is noteworthy that the addition of 1% and 1.5% dandelion elevated body weight in meat rabbits but decreased Firmicutes in the intestinal microflora. This phenomenon leads us to hypothesize that this discrepancy may be due to interspecies differences in digestive systems. The warty microflora is also able to elevate the level of glycolipid metabolism of the organism, which is a potential therapeutic mediator of glycolipid metabolism disorders (52); therefore, the increase in the level of *Micrococcus wartyi* in this study may be an important reason for the weight gain of meat rabbits, and its effect on the glycolipid metabolism of the digestive system of meat rabbits may be higher than that of the organisms of Micrococcus thick walled, and these phenomena also explain to a certain extent the phenomenon that there is not a positive correlation between the change in the level of Micrococcus thick walled and the increase in body weight of meat rabbits. However, relying solely on PICRUSt to predict microbial functions without functional verification and metagenomic analysis has certain limitations. For example, biases caused by insufficient database coverage and the inability to detect new functions or unknown genes. In the next step of the research, it may be necessary to add experiments such as multi-omics sequencing for further verification.

The results of genus-level analysis showed that microorganisms of the genus Akkermansia were elevated in the cecum of meat rabbits supplemented with 1% and 1.5% dandelion, and related studies of the genus Akkermansia have shown that there is a correlation between it and the homeostasis of glycolipid metabolism, so it is inferred that the addition of dandelion in the present study may have maintained the homeostasis of glycolipid metabolism in a certain way. In LEfSe analysis, the control group contributed more to the difference in bacterial community structure. Rigidiomycetes were found to be associated with infection of genotoxic *E. coli* strains in related studies (53), and Rigidiomycetes bacteria also appeared to be on the rise in the intestinal tracts of mice fed high-fat diets (54), which may indicate that Richenobacteriaceae are associated with a poor intestinal environment, such as intestinal inflammation. By contrast, for the 0.5% addition group, where the differences in bacterial flora structure contributed more to Clostridia, Clostridium spp. were found to be associated with a wide range of oral diseases, inflammatory bowel disease, intestinal infections, etc. (55). In addition, *Micrococcus wartyi* was the dominant strain in the 1.5% addition group, and Trichoderma sp. was the dominant strain in the 2% addition group, both of which are very common in the healthy intestines of animals.

### Conclusions

In conclusion, the addition of dandelion to rabbit diets can improve their meat quality, growth performance, and immune performance by enhancing their antioxidant and immune capacities, altering the quantity and composition of the intestinal microbiota, and protecting intestinal health to a certain extent. This provides new ideas for the healthy breeding of rabbits and the reduction of antibiotic use and ensures the provision of healthy and safe meat products for humans.

## Data Availability

The sequencing data have been provided with item numbers (PRJNA1009939) in the section Materials and Methods and can be searched inside NCBI.
